# Management and outcome of 239 adolescent and adult rhabdomyosarcoma patients

**DOI:** 10.1002/cam4.92

**Published:** 2013-07-15

**Authors:** Sarah N Dumont, Dejka M Araujo, Mark F Munsell, Jason A Salganick, Amaury G Dumont, Kevin A Raymond, Claude Linassier, Shreyaskumar Patel, Robert S Benjamin, Jonathan C Trent

**Affiliations:** 1 Hematology Oncology DepartmentSylvester Comprehensive Cancer Center, University of MiamiMiami, Florida; 2 Department of Sarcoma Medical OncologyMD Anderson Cancer Center, University of TexasHouston, Texas; 3 Department of BiotatisticsMD Anderson Cancer Center, University of TexasHouston, Texas; 4 Department of PathologyMD Anderson Cancer Center, University of TexasHouston, Texas; 5 Medical Oncology DepartmentBretonneau Hospital, Francois Rabelais UniversityTours, Loire Valley, France

**Keywords:** Adolescent, adult, age, multimodality treatment, rhabdomyosarcoma

## Abstract

Adult rhabdomyosarcoma (RMS) is a rare tumor that has inferior outcome compared to younger patient population. The present work aims to study the age-related differences in management of adolescents and adults with RMS. Under an institutional review board-approved protocol, we retrospectively analyzed 239 patients, 10 years of age and greater, diagnosed with RMS at MD Anderson Cancer Center from 1957 through 2003. Of the 239 patients, 163 patients were nonmetastatic with a median overall survival (OS) of 3.8 years (95% CI 2.8–7.6). In the multivariate analysis, age >50 was significantly associated with shorter OS and recurrence-free survival (RFS) for primary patients. Metastases were present in 76 patients, the median OS was 1.4 years. Approximately 13% of metastatic patients <50 years old had a long-term survival exceeding 15 years. Multimodality therapy, including surgery, radiotherapy, and chemotherapy was significantly associated with longer OS in primary and metastatic patients. Use of bi- and triple modality treatment decreased in metastatic patients over 50 years of age compared to younger patients. RMS in adolescents and adults has a poor outcome compared with younger individuals. Increased use of multidisciplinary therapy may improve older patient clinical outcome.

Adult rhabdomyosarcoma is a rare entity that has inferior outcome compared to younger patient population. This retrospective study emphasizes the age-related differences in management of patients that may partly explain their poor prognosis.

## Introduction

Rhabdomyosarcoma (RMS) is a rare mesenchymal tumor 1–2. It is the most common soft-tissue sarcoma in the first two decades of life, but accounts for <1% of adult cancers and is reported to account for <4% of adult soft-tissue sarcomas in the United States 3–4. Because cancer is fortunately a rare event during childhood, about four in 10 patients reported to have RMS are adults 5.

RMSs are classified into three main histologic subtypes: pleomorphic RMS (PRMS), embryonal RMS (ERMS), and alveolar RMS (ARMS). Classically, ERMS is correlated with a better prognosis than either ARMS or PRMS 6–11.

Some chromosome rearrangements are specific to a subtype and allow a finer classification of the tumor, thus having a prognostic importance 12–20. Therefore, the assessment of the translocation is becoming a tool for risk stratification in pediatric RMS 21–22 and is likely to have relevance for adult RMS as well 23.

The Intergroup Rhabdomyosarcoma Study (IRS) made the major observation that patients older than 10 years of age, defined in the present work as adolescent and adult patients, had inferior outcomes compared to patients younger than 10 11,22. The 5-year overall survival (OS) for adult patients with localized disease is a dismal 20–40% 6, whereas in pediatric patients it is between 60% and 80% 25. Five-year OS is <5% in adult patients with metastatic disease 3,26.

Because of its rarity, knowledge specific to patients over 10 with RMS comes mostly from cohort studies, explaining the fact that diagnosis and treatment strategy for adult patients is often emulated from the pediatric RMS guidelines 28.

Because several studies suggested that patients over 10 years of age experienced an inferior prognosis compared to younger children, the present work focuses on 239 patients, 10 years of age and greater, diagnosed with RMS at MD Anderson Cancer Center (MDACC).

We provide insight into the difference in outcome between adult and pediatric RMS patients, whether it is due to differences in biology itself through clinicopathologic description or differences in patient management.

## Patients and Methods

### Patients

We retrospectively analyzed 239 consecutive patients, 10 years of age and greater, diagnosed with RMS at MDACC from 1957 through 2003. Thirteen patients (5%) presented with relapse while the others received their primary treatment at our institution. We obtained a waiver of informed consent and the protocol was approved by the Institutional Review Board.

Patient demographics and tumor, treatment and survival data were reviewed. Tumors were classified as localized disease when they had not yet metastasized at the time of diagnosis. Locoregional lymph node involvement was included in the nonmetastatic group. Distant lymph node involvement was categorized as metastases.

The IRS staging system divides favorable from unfavorable sites and gives risk stratification management in pediatric RMS. The anatomic sites were at first clustered following this staging system but were broaden due to lack of prognostic significance. Tumor size was based on the largest dimension of the localized tumor as reported on pretreatment imaging scans.

At least two cycles of chemotherapy were required to be included in the treatment analysis.

### Pathology

Pathology review was performed at our institution by a sarcoma pathologist at the time of diagnosis for each patient and the specimens were rereviewed a second time at the inclusion to the study by a different sarcoma pathologist.

### Statistical analysis

Patient outcome was assessed according to the following clinicopathological variables: age, gender, size and location of localized tumor, nodal status, and IRS group classification. Treatment was analyzed according to the extent of multidisciplinary involvement as well as the type of chemotherapy agent.

The Kaplan and Meier product limit estimator estimated the median OS, recurrence-free survival (RFS) for nonmetastatic patients and progression-free survival (PFS) for metastatic patients from date of diagnosis to date of death or last follow-up. We used Cox proportional hazards regression to model OS, RFS, and PFS and to estimate the hazard ratios for several potential prognostic factors in a univariate fashion. We then included in a multivariate model all factors found to be significant at the 0.25 level and used backward elimination to remove factors until all remaining factors were significant at the 0.05 level. All statistical analyses were performed using SAS 9.1 for Windows (Copyright © 2002–2003 by SAS Institute Inc., Cary, NC).

## Results

### Patient demographics

**Figure 1:**
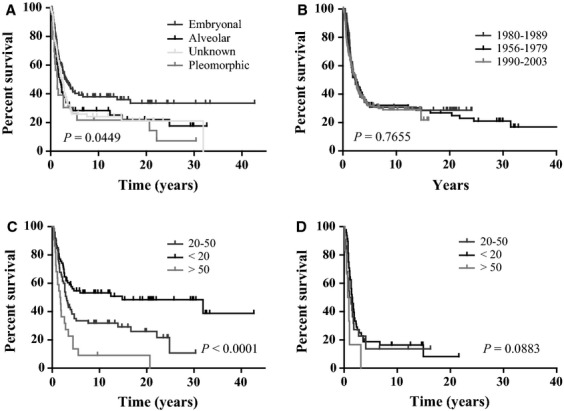
Kaplan–Meier patient overall survival comparing (A) histology subtypes, (B) era of treatment, (C) age in localized, and (D) metastatic setting.

The cohort included 239 patients, 10 years of age and older, followed and treated at our institution for a diagnosis of RMS. This represents only 1.2% of the 19,708 patients with sarcoma seen during the period of the study. The median age was 19 years with a range of 10–102, while 80% of patients were age 15 or older. There were 97 (40.6%) women and 142 (59.4%) men (Table 1). ERMS was the most represented subtype and tended to have a longer survival compared with other subtypes (Fig. 1A). To reflect the evolution of RMS management over the 45-year study period, survival was analyzed according to three different time periods: 1957–1979, 1980–1989, and 1990–2003. No difference in 5-year OS was observed between the three time periods and was stable around 33% (Fig. 1B). Moreover, incidence of nodal involvement was used as a surrogate for imaging improvement. Nodal detection was stable overtime with a rate of 7%.

**Table:** Patient and tumor characteristics

Description	No.	%
Gender		
Female	97	41
Male	142	59
Age (years)		
<20	122	51
20–50	88	37
>50	29	12
Subtype		
Alveolar	56	23
Embryonal	95	38
Pleomorphic	23	10
Unknown	65	27
Location		
Head and neck	87	36
GU	46	19
Trunk	23	10
Intraabdominal/pelvis	35	15
Extremities	48	20
IRS		
1	57	24
2	32	13
3	73	31
4	76	32
Unknown	1	0
IRS group		
I	52	22
II	13	5
III	88	37
IV	76	32
Unknown	10	4
Tumor size		
0–5 cm	88	37
5.01–10.00 cm	90	38
>10 cm	51	21
Unknown	16	7

GU, genitourinary; IRS, Intergroup Rhabdomyosarcoma Study.

### Localized disease

#### Patient demographics

163 patients (68%) had no evidence of metastases, including 63 (38.7%) women and 100 (61.3%) men. The median age was 22 with a range of 10–102. The mean age was 28.6 (standard deviation, 18.1).

#### Tumor characteristics

Sixty-two percent of the tumors were considered invasive at surgery or on imaging. The main tumor location was the head and neck (44%), followed by the genitourinary (GU) tract (20%) and the extremities (18%). The trunk and intraabdominal and pelvis locations each represented <10% of patients (Table 2).

**Table:** Factors associated with recurrence-free survival and overall survival for patients with localized disease

	No. of Pts (%)	Recurrence-free survival							Overall survival											
Median (years)		Uni variate *P*-value	Multivariate							Median (years)	Uni variate *P*-value	Multivariate											
			*P*-value	Hazard ratio	95% CI for HR	*P*-value	Hazard ratio	95% CI for HR			
Gender											
Female	63 (39)	2.6	–				4.4	–			
Male	100 (61)	1.4	0.142				3.3	0.396			
Age											
<20	74 (45)	6.7	–	–	1.00	–	31.9	–	–	1.00	–
20–50	66 (40)	1.4	0.003	0.010	1.77	1.15–2.73	3.0	0.006	0.036	1.78	1.04–3.03
>50	23 (14)	0.9	<0.001	<0.001	3.48	1.98–6.12	1.7	<0.001	<0.001	6.02	2.92–12.42
Location											
Head and neck	72 (44)	1.5	–				3.0	–			
GU	33 (20)	13.9	0.039				–	0.030			
Trunk	14 (9)	1.0	0.630				2.4	0.708			
Abdominal/pelvis	14 (9)	1.3	0.716				3.2	0.519			
Extremities	30 (18)	2.2	0.617				3.4	0.611			
IRS											
1	57 (35)	3.9	–				14.8	–			
2	32 (20)	2.0	0.826				7.6	0.628			
3	72 (44)	1.2	0.051				2.6	0.004			
Unknown	1 (1)	–	–				–	–			
IRS group											
I	52 (32)	1.9	–				13.9	–			
II	13 (8)	2.4	0.969				2.9	0.657			
III	87 (53)	1.7	0.652				3.3	0.166			
Unknown	10 (6)	–	–				–	–			
Chemotherapy											
Doxorubicin, no ifosfamide	63 (39)	1.7	–				2.8	–			
Ifosfamide, no doxorubicin	5 (3)	1.3	0.376				4.2	0.954			
Doxorubicin plus ifosfamide	19 (12)	1.2	0.095				2.1	0.192			
Actinomycin D, no doxorubicin/ifosfamide	33 (20)	–	0.015				–	0.002			
Any regimen, no actinomycin D/doxorubicin/ifosfamide	2 (1)	1.3	0.249				1.8	0.168			
Sequential doxorubicin/ifosfamide	6 (4)	5.0	0.687				9.5	0.891			
No chemotherapy	17 (10)	0.6	<0.001				2.8	0.323			
Unknown	18 (11)	3.1	–				3.4	–			
Treatment modality											
Chemotherapy	10 (6)	0.9	–				1.1	–	–	1.00	–
Surgery or surgery plus radiotherapy	15 (9)	0.5	0.113				2.9	0.417	0.300	0.59	0.21–1.61
Surgery plus chemotherapy or chemotherapy plus radiotherapy	58 (36)	5.9	0.120				–	0.013	0.043	0.41	0.17–0.97
Surgery plus chemotherapy plus radiotherapy	60 (37)	2.0	0.441				3.3	0.108	0.074	0.47	0.20–1.08
Unknown	20 (12)	–	–				–	–	–		
Tumor Size											
0.00–5.00 cm	67 (41)	2.4	–				4.6	–			
5.01–10.00 cm	61 (37)	1.6	0.998				4.4	0.645			
>10.00 cm	28 (17)	1.5	0.627				3.1	0.285			
Unknown	13 (8)	–	–				–	–			

GU, genitourinary; IRS, Intergroup Rhabdomyosarcoma Study.

Tumors measured most often <5 cm (41%); whereas 37% were 5–10 cm and 17% were larger than 10 cm. Tumor size was not specified in 8% of cases.

#### Treatment

**Figure 2:**
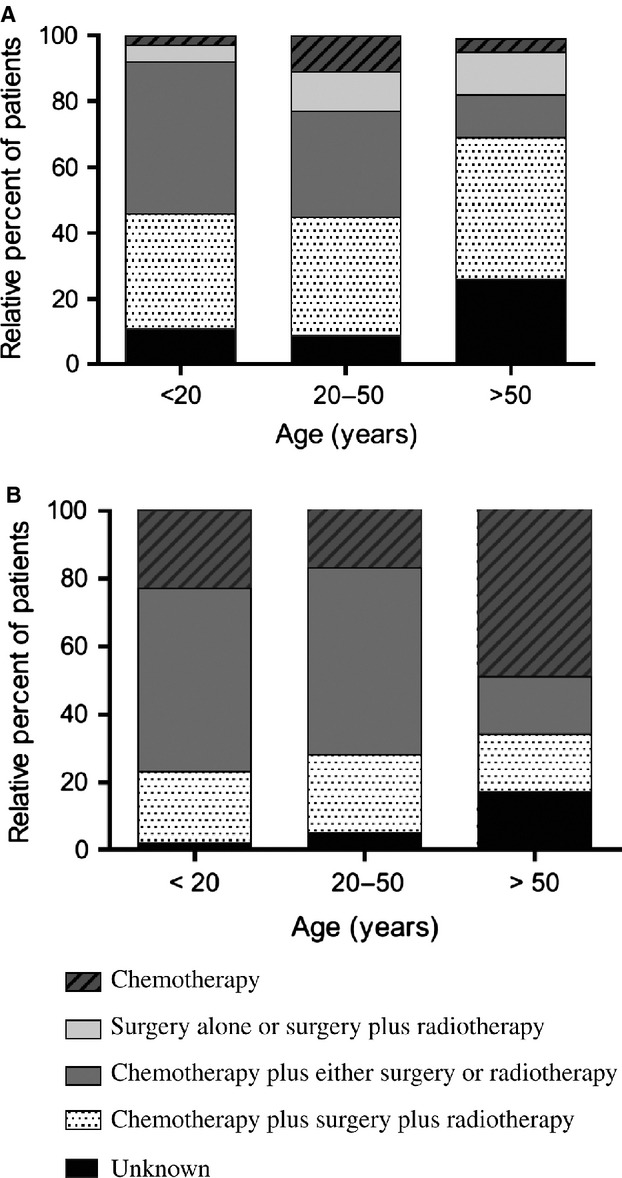
Treatment modality for (A) localized patients (B) metastatic patients.

Very few patients underwent localized therapy alone (20 patients, 9%) while 6% (14 patients) had chemotherapy alone. Many patients had multimodality therapy integrating surgery, chemotherapy, and radiotherapy (37%) whereas 36% had chemotherapy-based therapy with either surgery or radiotherapy. Patients over 50 years of age were more likely to have multimodality therapy compared with younger patients but the rate of chemotherapy-based strategies was fairly similar over age in the localized patient population (Fig. 2A). A chemotherapy regimen that included actinomycin D was given to 23% of patients. The patients receiving actinomycin D were almost exclusively (91%) under 20 years old. Doxorubicin was administered to 54% of patients and ifosfamide to 18% of patients. Eighty percent of patients receiving doxorubicin plus ifosfamide were between 20 and 50 years of age. Patients over 50 were less likely to receive ifosfamide than patients between ages 20 and 50 (Table 3). Ninety-nine percent of patients with localized RMS received at least one of the three drugs.

**Table:** Chemotherapy regimens according to age in localized and metastatic setting

Disease stage	Localized							Metastatic												
Age	<20			20–50					>50							<20									20–50											>50												
Chemotherapy regimen	No.	%	No.	%	No.	%	No.	%	No.	%	No.	%
Ifosfamide-free regimen												
Any doxorubicin, no ifosfamide	26	35	27	41	14	48	22	46	12	55	4	67
Any actinomycin D, no doxorubicin/ifosfamide	30	41	1	2	2	7	13	27	0	0	0	0
Any regimen, no actinomycin D/doxorubicin/ifosfamide	0	0	2	3	0	0	1	2	1	5	0	0
Ifosfamide-based regimen												
Any ifosfamide, no doxorubicin	3	4	2	3	0	0	4	8	1	5	0	0
Any ifosfamide plus doxorubicin	1	1	16	24	3	10	4	8	6	27	1	17
Sequential doxorubicin plus ifosfamide	2	3	4	6	0	0	3	6	1	5	0	0
No chemotherapy	4	5	9	14	4	14	0	0	0	0	0	0
Unknown	8	11	5	8	6	21	1	2	1	5	1	17
Total	74	100	66	100	29	100	48	100	22	100	6	100

#### Outcome

The analyses of RFS and OS are summarized in Table 2. One hundred twelve (69%) of the 163 patients had recurrent disease or died. Median follow-up for all 163 patients with localized disease was 3.3 years (range, 0.3–42.7 years). The 64 patients who remained alive had a median follow-up of 12.1 years (range, 0.6–42.7 years) while the 99 patients who died had a median follow-up of 1.8 years (range, 0.3–31.9 years).

The median RFS was 1.9 years (95% CI 1.3–2.8 years), the 1-year RFS rate was 0.67 (95% CI 0.59–0.73), the 2-year RFS rate was 0.469 (95% CI 0.390–0.544), and the 5-year RFS rate was 0.362 (95% CI 0.288–0.436). Thirteen patients had recurrent disease but remained alive at last follow-up. In the univariate analysis of RFS, increasing age, and “No Chemotherapy” were associated with shorter RFS, while GU location and “Any actinomycin D, No doxorubicin/ifosfamide” were significantly associated with longer RFS. Patients who did not receive chemotherapy had a complete resection with or without radiation therapy. However, in the multivariate analysis only an increasing age was associated with a shorter RFS (Table 2).

The median OS for all patients with localized disease was 3.8 years with 95% confidence interval (CI) 2.8–7.6 years. Ninety-nine of the 163 patients died. The 1-year OS was 0.846 with 95% CI 0.781–0.893. The 2-year OS was 0.660 with 95% CI 0.582–0.727, and the 5-year OS was 0.441 with 95% CI 0.362–0.516. In the univariate analysis of OS, increasing age and IRS grade 3, were significantly associated with shorter OS, while GU location, “any actinomycin D, no doxorubicin/ifosfamide”, and “surgery plus chemotherapy or chemotherapy plus radiotherapy” were significantly associated with longer OS. In the multivariate analysis only increasing age and inclusion of chemotherapy (“surgery plus chemotherapy or chemotherapy plus radiotherapy”) were associated with longer OS (Table 2). The Kaplan–Meier survival curve presented a notable inflection point at 5 years followed by a plateau. Long-term survivors (>15-year) included 55% of patients younger than 20, 31% of patients between 20 and 50 years of age, but <10% of patients older than 50 (5-year OS 13%, median OS of 1.7 years, Fig. 1C).

### Metastatic disease

#### Patient demographics

There were 76 patients with metastatic disease, including 34 (44.7%) women and 42 (55.3%) men. The median age was 18 (10–67 years). The mean age was 23.7 (standard deviation, 13.9).

#### Tumor characteristics

Localized tumors were found within the abdomen or pelvis in 28%, the extremity in 24% of the tumors were located in the extremities, the head and neck in 20%, in the GU region in 17%, and on the trunk in 12% of patients. Two thirds of the primary tumors measured >5 cm (68%) at diagnosis (Table 4). The main metastatic sites were the lungs in 39% of cases, the bone marrow for 28%, distant lymph nodes in 20%, and the bone for 14% of patients.

**Table:** Factors associated with progression-free survival and overall survival for patients with metastatic disease

	No. of Pts (%)	Progression-free survival							Overall survival										
Median (years)		Univariate *P*-value	Multivariate							Univariate *P*-value	Multivariate										
			*P*-value	Hazard ratio	95% CI for HR	*P*-value	Hazard ratio	95% CI for HR		
Gender										
Female	34 (45)	0.8	–				–			
Male	42 (55)	1.0	0.541				0.672			
Age										
<20	48 (63)	1.1	–	–	1.00	–	–	–	1.00	–
20–50	22 (29)	0.7	0.384	0.011	2.44	1.23–4.87	0.428	0.015	2.44	1.19–5.00
>50	6 (8)	0.5	0.037	0.139	2.10	0.79–5.61	0.036	0.158	2.02	0.76–5.36
Location										
Head and neck	15 (20)	1.8	–	–	1.00	–	–	–	1.00	–
GU	13 (17)	1.4	0.703	0.187	1.93	0.73–5.16	0.718	0.220	1.93	0.67–5.56
Trunk (chest wall/back)	9 (12)	1.0	0.035	0.013	4.00	1.35 –11.85	0.014	0.006	5.06	1.60–16.02
Intraabdominal/pelvis	21 (28)	0.6	0.001	0.002	4.17	1.73 –10.08	0.002	0.003	4.02	1.61–10.05
Extremities	18 (24)	0.8	0.073	0.008	3.61	1.40–9.36	0.072	0.010	3.90	1.39–10.96
IRS										
3	1 (1)	–	–				–			
4	75 (99)	1.0	–				–			
IRS group										
III	1 (1)	–	–				–			
IV	75 (99)	1.0	–				–			
Chemotherapy										
Doxorubicin, no ifosfamide	38 (50)	1.0	–				–			
Ifosfamide, no doxorubicin	5 (7)	1.4	0.654				0.404			
Doxorubicin plus ifosfamide	11 (14)	0.6	0.201				0.390			
Actinomycin D, no doxorubicin/ifosfamide	13 (17)	0.9	0.792				0.750			
Any regimen, no actinomycin D/Doxorubicin/Ifosfamide	2 (3)	1.3	0.815				0.808			
Sequential doxorubicin/ifosfamide	4 (5)	1.7	0.898				0.251			
Unknown	3 (4)	0.8	–				–			
Treatment modality										
Chemotherapy	18 (24)	0.6	–	–	1.00	–	–	–	1.00	–
Surgery plus chemotherapy or chemotherapy plus radiotherapy	39 (51)	1.0	0.051	0.131	0.60	0.32–1.16	0.059	0.060	0.52	0.27–1.03
Surgery plus chemotherapy plus radiotherapy	16 (21)	1.7	0.003	0.037	0.40	0.17–0.95	0.003	0.018	0.34	0.14–0.83
Unknown	3 (4)	–	–	–	–	–	–			
Tumor Size										
0.00–5.00 cm	21 (28)	1.3	–				–			
5.01–10.00 cm	29 (38)	1.0	0.447				0.404			
>10.00 cm	23 (30)	0.5	0.142				0.172			
Unknown	3 (4)	–	–				–			

GU, genitourinary; IRS, Intergroup Rhabdomyosarcoma Study.

#### Treatment

All the patients received chemotherapy. Thirty-nine percent had either surgery or radiotherapy associated with chemotherapy while 21% had all three modalities. Seventeen percent of patients received actinomycin D, 70% doxorubicin, and 26% ifosfamide. Only two patients received none of these three agents (3%). Patients with metastatic disease had a 14.4% 15-year survival treated with bimodality treatment (chemotherapy plus surgery or chemotherapy plus radiotherapy) and patients who were able to be treated with all three modalities (surgery plus chemotherapy plus radiotherapy) had a 37.5% 14-year survival. Use of surgery and/or radiation therapy in addition to chemotherapy decreased in metastatic patients over 50 years of age compared to younger patients (Fig. 2B), and their chemotherapy was less likely to include ifosfamide than patients between 20 and 50 years of age (Table 3).

#### Outcome

The analyses of PFS and OS are summarized in Table 4. Median follow-up for all 76 patients with metastatic disease was 1.4 years (range, 0.1–21.6 years). For the 14 patients who remain alive the median follow-up was 8.9 years (range, 1.1–21.6 years). For the 62 patients who died the median follow-up was 1.1 years (range, 0.1–14.9 years).

The median PFS was 0.9 years (95% CI 0.7–1.3 years). Sixty-seven (88%) of the 76 patients had progressive disease or died. The PFS rate at 1-year was 0.447 (95% CI 0.334–0.555), at 2-years was 0.224 (95% CI 0.138–0.322), and at 5-years was 0.132 (95% CI 0.067–0.218). Five patients with progressive disease remained alive at last follow-up. In the univariate analysis age >50 and location in the trunk or abdomen/pelvis region were significantly associated with shorter PFS, while patients who were able to receive surgery, radiation, and chemotherapy were associated with longer PFS than those who did not. In the multivariate analysis these same factors were found to be associated with PFS, along with location in the extremities (Table 4).

The median OS for all patients with metastatic disease was 1.4 years (95% CI 1.0–1.8 years). The median OS at 1-year was 0.605 (95% CI 0.486–0.705), at 2-years was 0.323 (95% CI 0.221–0.429), and at 5-years was 0.180 (95% CI 0.102–0.277).

In the univariate analysis of OS, age >50 and location in the trunk or abdomen/pelvis were associated with shorter OS, while patients who were able to receive surgery, radiation, and chemotherapy were associated with longer OS compared with those who did not. These three factors remained significant in the multivariate analysis of OS in patients with metastatic disease (Table 4).

In the multivariate analysis of OS, location in the extremities was also found to be associated with a longer PFS. The Kaplan–Meier curve shows an inflection point around 3 years, followed by a plateau for patients younger than 50 suggesting a cure rate of ~17% for these metastatic patients (Fig. 1D).

## Discussion

Adolescent and adult RMS is a rare entity that has inferior outcome compared to younger patient population. Our study shows not only that this patient population have a similar clinicopathologic pattern than pediatric RMS, but also that adolescent and adult patients before 50 years of age are more likely to receive multidisciplinary therapy and ifosfamide-based chemotherapy than their older counterparts.

The first major difference between pediatric and older patients is the referral pattern. Indeed, pediatric cancers are most likely to be referred to tertiary or quaternary cancer center, thus benefiting from a more accurate diagnosis, which is critical in this disease. According to the Surveillance, Epidemiology and End Results (SEER) study comparing adult and pediatric RMS patients between 1973 and 2005 for a total of 2600 patients, the histologic subtype was unknown for about 43% of adult patients versus 13.2% of pediatric patients 5. SEER data reflecting the average population management, we could assume that in the early 70s, many malignant fibrous histiocytoma were mistaken for PRMS by nonsarcoma-trained pathologists. MDACC being a referral center for sarcoma treatment, about 27% of pathology specimens could not be characterized, particularly from patients treated during the earliest time period, which suggests that referring adult RMS patients to an expert center increases the chances of a better characterization of the disease 29.

Moreover, our study survival data reveals two major observations. First, patients with metastatic RMS were found to have an 18% survival rate 5 years from diagnosis with an apparent 12% survival past 15 years, which is unexpectedly high in this setting. Second, patients older than 50 years with localized RMS have a dismal 5-year OS of 13%. As this finding could not merely be explained by the natural aging of patients, metastatic patients of this age group did not share the similar long-term survival trend. This raises the question that patients over 50 years of age with localized disease may be treated less effectively than the younger patients.

While looking closer at the treatment disparities among age groups, the first aspect was that, in localized disease, the probability of not receiving chemotherapy increased with age, which may have reduced the probability of long-term survival. Meanwhile, patients older than 50 years of age with metastatic RMS were much more likely to receive chemotherapy only whereas their younger counterparts were more likely to be treated with approaches including surgery or radiotherapy. This finding confirms the recent study from Kojima et al. 30 that judicious use of local therapy is critical to survival of patients with metastatic disease. Moreover, chemotherapy regimens including ifosfamide are the mainstay of chemotherapy used in older patients with RMS; however, ifosfamide is often withheld or limited in its use due to associated toxicity in this population. This leads to speculation that perhaps alternative agents such as cyclophosphamide could be used more frequently.

Alternatively, RMS is possibly a different biological and clinical entity in patients aged 10 years or greater. As the subtype and translocation status have prognostic significance, the different pattern of histology across age, for instance the pleomorphic subtype uncommon in pediatric patients, may explain the difference in outcome. While few studies focused on the age-related biological and molecular differences of RMS 23, the cell of origin of RMS was investigated in recent works such as the European Pediatric Soft Tissue Sarcoma Study Group showing that the patient outcomes and gene expression signatures of fusion-negative ARMSs were very similar to those of ERMSs, and may help better understand the relationship between outcome and histological subtype 31.

Finally, a report based on SEER data showed that the outcome of children and adolescent younger than 15 years of age with RMS appeared to have improved between 1975 and 1982, reflecting the impact of management improvement as a result of clinical trial participation but had been stable since 32.

Possibly due to methodology, selection bias, or referral patterns, our work shows no quantifiable clinical improvement in the outcome of older patients over the last 50 years despite a better understanding of the disease biology, imaging, and treatment progress.

Despite its inherent limitations, this study highlights the need to improve the management of patients with RMS over 10 years of age. We should ensure that these patients benefit as much from clinical management and research progress as younger patients with RMS.

## Conflict of Interest

None declared.
